# Applying Social Network Analysis to Evaluate Implementation of a Multisector Population Health Collaborative That Uses a Bridging Hub Organization

**DOI:** 10.3389/fpubh.2018.00315

**Published:** 2018-11-02

**Authors:** Aaron L. Leppin, Janet M. Okamoto, Paige W. Organick, Anjali D. Thota, Francisco J. Barrera-Flores, Mark L. Wieland, Rozalina G. McCoy, Robert P. Bonacci, Victor M. Montori

**Affiliations:** ^1^Knowledge and Evaluation Research Unit, Division of Health Care Policy and Research, Mayo Clinic, Rochester, MN, United States; ^2^Department of Health Sciences Research, Mayo Clinic, Scottsdale, AZ, United States; ^3^Knowledge and Evaluation Research Unit, Mayo Clinic, Rochester, MN, United States; ^4^Division of Endocrinology, Universidad Autonoma de Nuevo Leon, Monterrey, Mexico; ^5^Division of Primary Care Internal Medicine, Mayo Clinic, Rochester, MN, United States; ^6^Division of Primary Care Internal Medicine, Division of Health Care Policy and Research, Mayo Clinic Robert D. and Patricia E. Kern Center for the Science of Health Care Delivery, Mayo Clinic, Rochester, MN, United States; ^7^Department of Family and Community Medicine, Mayo Clinic, Rochester, MN, United States

**Keywords:** social network analysis, population health, health promotion, community based programs, partnerships

## Abstract

**Background:** Multisector collaboratives are increasingly popular strategies for improving population health. To be comprehensive, collaboratives must coordinate the activities of many organizations across a geographic region. Many policy-relevant models encourage creation and use of centralized hub organizations to do this work, yet there is little guidance on how to evaluate implementation of such hubs and track their network reach. We sought to demonstrate how social network analysis (SNA) could be used for this purpose.

**Methods:** Through formative research, we defined and conceptualized key characteristics of a bridging hub network and identified a set of candidate measures**—**(1) network membership, (2) network interaction, (3) role and reach of the bridging hub, and (4) network collaboration—to evaluate its implementation within a pre-determined geographic region of Southeast Minnesota, USA. We then developed and administered a survey to assess outcomes as part of a SNA. We commented on the feasibility and usefulness of the methods.

**Results:** The initial surveyed network consisted of 50 healthcare organizational sites and 50 community organizations representing sectors of public health, education, research, health promotion, social services, and long-term care and supports. Fifty-three of these organizations responded to the survey. The network's level of collaboration was “Cooperation” (level 2 of 5) and reported levels of collaboration varied by organization. Thirty-eight additional, unsurveyed organizations were identified as collaborators by respondents, pushing the theoretical network denominator up to 138 organizations. These additional organizations included grocery stores, ambulance services, and smaller, independent healthcare and community-based services focused on meeting the needs of underserved populations. The bridging hub organization had the highest betweenness centrality and was in good position to bridge healthcare and the community, although its organizational reach was estimated at only 51%. The SNA methods were feasible and useful for identifying opportunities and guiding implementation.

**Conclusions:** Bridging hub organizations are not likely to link—or even be aware of—all relevant organizations in a geographic region at initial implementation. SNA may be a useful method for evaluating the value and reach of a bridging hub organization and guiding ongoing implementation efforts.

**Trial registration: **http://ClinicalTrials.gov; #NCT03046498

## Background

### Social networks and population health

The overall health of individuals and communities results from the complex interplay of many determinants. Behavioral, social, and environmental factors account for most of a population's potential for health, while health care—and the systems in place to deliver it—contribute only 10–20% (1, 2). Models aimed at optimizing population health encourage the clinical integration and support of community-based partners well-positioned to influence the myriad of factors that make health possible (3–7). To integrate care and services practically, multi-sector collaboratives must often be developed.

The public health literature is replete with examples of such collaboratives and has proposed social network analysis (SNA) as a useful methodology for evaluating their strengths and structure (8). SNA is a quantitative and visual method for studying social relationships (9). In 2008, Varda and colleagues provided guidance on how to use SNA to evaluate public health collaboratives by considering two theory-based strategies and operationalizing seven measures of collaborative connectivity (10). These methods have been used to consider the dimensions and describe the structures of several health networks serving a variety of purposes (11–15). Despite its many advantages, SNA has not been explicitly refined to direct and evaluate the proactive implementation of key policy relevant models aimed at improving population health.

### Bridging hub networks

For the purposes of orienting this paper, we will define a subset of these policy relevant models as “bridging hub networks.” The overarching goal of bridging hub networks as we conceptualize them is to make geographic areas accountable for the overall health of their populations. To realize this goal, bridging hub networks prioritize the development of a new entity—a bridging hub organization—that can efficiently coordinate and bridge services across sectors. Bridging hub organizations are similar in function to network administrative organizations (16), although they use a shared governance model intended to support the collective impact of network members.

The CMS Innovation Center (CMMI)'s Accountable Health Communities (AHC) model—currently being tested in 32 communities in the United States (17)—represents one example of a potential bridging hub network. In the AHC model, clinical sites are expected to screen for health-related social needs among all Medicare and Medicaid beneficiaries and facilitate referral and linkage to community-based services. The most mature manifestation of this model (the so-called Alignment Track) includes the creation of a “backbone organization” (e.g., a bridging hub organization) that is overseen by an advisory board and is focused on building community capacity, sharing data, and improving the quality of services.

The Pathways Community HUB (HUB) Model—developed by Drs. Mark and Sara Redding and promoted by the Agency for Healthcare Research and Quality (AHRQ)—is conceptually similar to the AHC model in that it aims to engage at-risk individuals and connect them to valuable services (18). It differs, however, in that it is more disease-focused and uses community-based care coordinators to initiate “Pathways” that match interventions and services to individuals' risk factors. This allows for tracking of activity and the development of payment agreements that pay for the completion of Pathways. In the HUB model, a neutral and independent “HUB” organization (again—a potential bridging hub organization) is responsible for coordinating a network of agencies and care coordination services and administering contracts and activities across a defined region.

Finally, in an effort to increase the translation of evidence-based programs and interventions that support healthy aging, the United States Administration on Community Living (ACL) has funded multiple initiatives aimed at increasing communities' capacity to deliver services through “hub-based, integrated networks.” Specifically, the National Council on Aging leads a Network Development Learning Collaborative (19) and the National Association of Area Agencies on Aging coordinates an Aging and Disability Business Institute (20). Both of these ACL-funded efforts are targeted toward region and state level colllaboratives aiming to develop community-based networks that can partner with healthcare delivery organizations and payers.

#### Objective

The objective of this paper is to illustrate—through an example from our own research—how SNA can be used to evaluate progress in the implementation of population health models (such as the AHC, HUB, and ACL models) that fit the definition of a bridging hub network. We begin by describing the context in which we were trying to implement our model and its purpose. We then present the rationale for the SNA metrics we adopted to evaluate implementation, along with our methods and results. We conclude by providing lessons learned and suggestions for practice, policy, and research going forward.

#### Our setting

In 2015, our research group conducted a community-engaged, participatory research project focused on identifying barriers and opportunities related to scaling and clinically integrating evidence-based health promotion programs offered in community settings. The research was focused on an 11-county region of Southeast Minnesota (~15,000 square miles) that is mostly rural, but included a centrally located city with a population of 110,000. The study's primary conclusion [details are published elsewhere (21)] was that a systems-based approach would be helpful in reorganizing the community, building its capacity for service provision, and streamlining its connections with healthcare. Consequently, our community-partnered research team sought to create a “community system for well care” to coordinate health promotion activities across many organizations and to partner with and complement healthcare systems across the region.

To operationalize this system, we created an independent collaborative entity. Our intention was that this “bridging hub” would serve as an administrator and connector that could organize the health promotion programming of many community based organizations under a single, parent brand. To equitably fulfill its purpose, we felt the hub would need to connect a network that reached all corners of the geographic region and served all the sub-populations and health systems it comprised. In January of 2016 the collaborative adopted the moniker of the WellConnect Southeast Minnesota Partnership (WellConnect) and in July of that year it convened its first official Steering Committee. Over the next year, WellConnect sought to grow into the role of a bridging hub organization by engaging what it perceived to be relevant organizations, commissioning the building of a coordinating technology, and adopting and promoting a single brand identity. The web-based technology was launched in September of 2016 with an intent of mediating activity across the network. One month later, we received research funding to evaluate through SNA the relationships between health-related organizations in Southeast Minnesota with a focus on the role and position of WellConnect at that time. The project was approved by the Institutional Review Boards of Mayo Clinic and Olmsted Medical Center and registered on Clinical Trials.gov (NCT03046498).

#### Selection of measures

**Figure 1:**
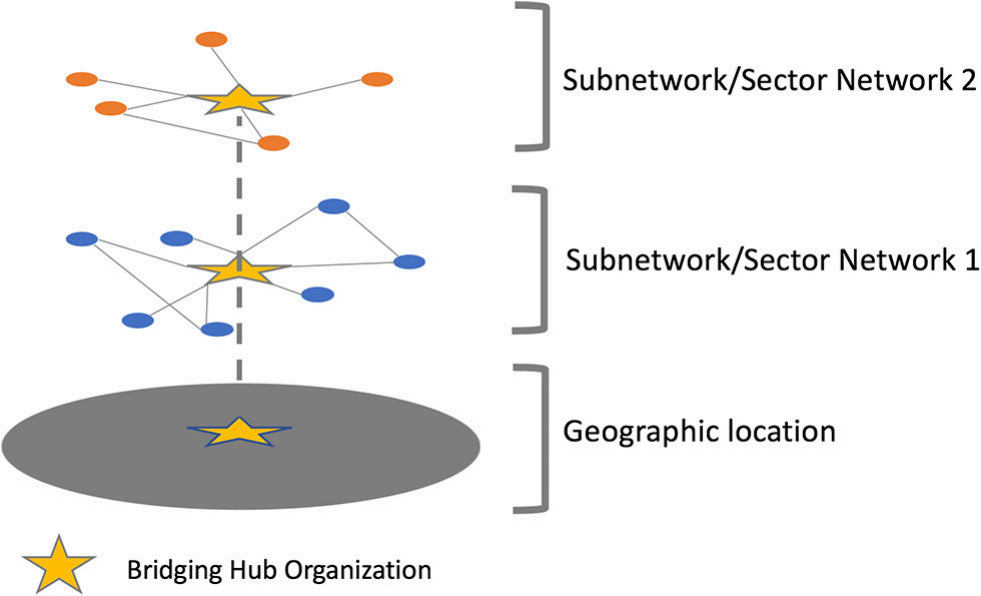
A theoretical bridging hub network linking two subnetworks through a bridging hub organization.

Through a series of discussions and meetings with stakeholders and among our research team, we translated ideas about what a bridging hub network should look like—and the functions it should perform—into a theoretical network structure. We then identified a set of metrics—drawing heavily on the work of Varda et al. (10)—that would be practically useful in evaluating its implementation and reach. We adapted the structural holes theory (22) to ground the network's design because (1) its aim to bridge subnetworks (across sectors and geography) by filling “structural holes” is consistent with the development of a bridging hub organization and (2) networks developed in this way limit weak ties and streamline communication through a mediator in a way that is more efficient and resource-conscious. Our prior work (21) had also suggested the existence of current, unbridged subnetworks within healthcare and the community (e.g., by system and sector, respectively) that would be conducive to this design. For a theoretical representation of a bridging hub network see Figure 1. For a summary of measures we adopted to evaluate its implementation, see Table 1.

**Table 1:** Proposed social network analysis (SNA) measures for evaluating implementation of bridging hub networks.

**Network characteristic**	**Description**	**Metrics**
Network membership	Organizations in (and out of) the network and their characteristics	Number and names of organizations in network, including their type, sector/mission, size
Network interaction	Patterns and positions of network members in relation to achievement of the model	Location of organizations over geography and the ties that connect them, including presence and types of subnetworks
Role and reach of the bridging hub	Special consideration of the role of the bridging hub organization in linking subnetworks and in achieving complete organizational reach/comprehensiveness	Measures of in-degree and betweeness centrality and proportions of organizations linked to the bridging hub and/or number of subnetworks bridged
Network collaboration	Evidence of purposeful interaction built on trust and common goals	Measures of collaboration or frequency of interactions, trust and reciprocity of communication or a combination thereof

## Methods

### Sna measure development

In the spring of 2017 we developed a single survey measure that would allow us to assess all key characteristics of a bridging hub network while also discovering potential network members we were unaware of (this was intended to help us estimate the baseline organizational reach of the hub). We based the measure on the Levels of Collaboration Survey (23), a survey that asks respondent organizations to rate their level of collaboration with other organizations. Its constructs were based on a review of collaboration literature, and its reliability and sensitivity to change were confirmed among organizations collaborating in grants (23). Possible levels on the scale are “no interaction at all,” “networking,” “cooperation,” “coordination,” “coalition,” and “collaboration.” To orient respondents to the scale and help guide selection, all surveys were administered with accompanying definitions and examples of the levels (see Table 2). We prefaced the instrument with instructions that the survey could be completed by an individual or by a group so long as the responses represented the perceptions of the organization as a whole, and with a request for respondents to “rate the extent to which your organization collaborates with the following organizations to improve health in Southeast Minnesota.” All surveys included language indicating that participation was voluntary and interpreted as consent for research.

**Table 2:** Possible levels of collaboration among organizations.

**Level option**	**Definition**	**SNA weight**
No interaction at all	No interaction, not aware	0
Networking	Aware of organization, loosely defined roles, little communication, all decisions made independently	1
Cooperation	Provide information to each other, somewhat defined roles, formal communication, all decisions made independently	2
Coordination	Share information and resources, defined roles, frequent communication, some shared decision making	3
Coalition	Share ideas and resources, frequent and prioritized communication, all members have a vote in decision making	4
Collaboration	Members belong to one system, frequent communication is characterized by mutual trust, consensus is reached on all decisions	5

### Participants

To populate the list of organizations against which respondents would rate their level of collaboration, we began by listing all healthcare clinical sites (noting that there could be many individual sites within a given integrated system) and all county public health offices across the region. We then added known community organizations across multiple sectors and conducted internet searches and queried stakeholders to identify additional organizations (because it was not the focus of this project, we did not include organizations focused solely on child health and wellbeing). After reaching ~85 organizations, it became increasingly difficult to identify additional organizations. For this reason and in order to keep the measure concise, we stopped when we reached 100 organizations. For all surveys, we developed organization-specific cover letters and marked out appropriate response rows to ensure that organizations could not rate their level of collaboration with themselves. As such, we asked each of the 100 organizations to rate its level of collaboration with each of the other 99. To identify important organizations we were not aware of, we included open-ended items at the end of the measure that asked respondents to list up to three additional organizations that they collaborated with that we did not ask about. Respondents were not asked to rate their level of collaboration with these organizations.

### Data collection

We administered all surveys in pen and paper format with postage paid return envelopes via postal mail or hand delivery in the Summer of 2017. We contacted non-respondents as feasible and kept track of reasons for non-participation when known. We entered data from completed surveys by study ID into a secure Excel relational data matrix (available in Supplementary Material), linking respondents to their reported collaborators, and double-checking for accuracy. We assigned values of 0–5 for the possible levels of collaboration (as described above), counting missing data from otherwise completed surveys as 0 (with the exception of the row representing self). We also developed and populated an accompanying attribute file of organizational characteristic data for each of the organizations using publicly available information. Attributes included were coded values representing the organizations' identity (ID), mission (sector), geographic population of focus (individual counties vs. entire region), position within the healthcare system vs. community, and—if in healthcare—healthcare system affiliation vs. independent.

### Analysis

We conducted the network analysis with Gephi software (24). To permit exploration of discrepancies in perception of collaboration, we considered all ties directed. We counted missing data as a null tie. To assess the connectedness of organizations, we prioritized in-degree centrality (e.g., a measure of incoming ties, or connections). We chose this measure (as opposed to out-degree centrality) because it allowed us to represent organizations that did not provide data and because it helped us to avoid over-representing organizations who reported a lot of outgoing ties. Specifically, we sought to emphasize the importance of organizations that peers reported as important collaborators rather than those that self-reported high levels of collaboration. We explored the data visually, via algorithm-generated network diagram, by coding the various attributes, and by arranging nodes manually to represent the actual geography. To explore outcomes of network membership, we compiled a list of network members overall, by sector, and by county/location and looked for evidence of isolates, or unconnected nodes. We calculated the number and prevalence of organizations listed in the open text fields (e.g., those that could play a role in the network but we were unaware of) and estimated the effect of their exclusion on key insights. To assess network interaction, we looked for the presence of subgroups by all attributes and by geography—mapping all organizations to their geographic location. To assess the role of WellConnect (the bridging hub organization), we calculated in-degree and betweenness centrality (a measure that assesses the number of shortest paths that go through or are “bridged” by a given node) for all organizations and explored the relative position of the bridging hub. To estimate hub reach, we also calculated the proportion of organizations overall and by sector that were linked to the bridging hub with and without inclusion of the organizations mentioned in open text fields. Measures of collaboration were factored into the weighting of all relationships by design of the survey measure and we supplemented this evaluation by searching for evidence of reciprocity (e.g., mutual agreement of the presence or absence of network interaction) and trust (e.g., frequencies of high levels of collaboration in organizational linkages) within and across subgroups and especially with the bridging hub.

## Results

### Participants

We received survey responses from 54 organizations (54%). Non-responders were more likely to be from the healthcare sector (*n* = 28) than from the community (*n* = 18). From returned surveys, data was missing in 8, accounting for < 1% of all cells. Twenty-nine (53%) of the responding organizations listed one or more additional organizations that they collaborated with that were not queried, summing to 38 additional unique organizations in total.

### Network membership

Network membership was complete in that all 100 organizations surveyed (even those not reporting data) were linked to at least one other organization. Half (50) of the organizations comprised the healthcare sector and these were distributed across six integrated healthcare systems and seven independent practices. The healthcare systems varied in size from 2 to 18 organizational sites. The remaining 50 community organizations broadly represented sectors of public health (*n* = 11), long-term services and care (*n* = 8), education (*n* = 5), health promotion (*n* = 11), research (*n* = 2), and social services (*n* = 11). Most of the community organizations focused on supporting health at the community or county level, although 15 had a regional focus. Two community organizations were described as “integrators” although WellConnect was the only organization with the explicit mission of bridging sectors across the entire region. The 38 additional organizations identified by respondents were not added to the network map but were noted to be predominantly community-based organizations in more peripheral parts of the region (74% of the organizations were from rural areas). Importantly, many of these additional organizations were smaller, independent entities with less formal connections to established integrated systems. Some utilized volunteers to provide long-term supports. The eight additional healthcare sites identified were also mostly small, independent organizations; most focused on providing free or low cost care to disadvantaged and minority populations, including mental health services. Respondents also reported collaboration with grocery (*n* = 3 organizations) and ambulance/emergency response (n−3 organizations) sectors in ways we did not predict.

### Network interaction

**Figure 2:**
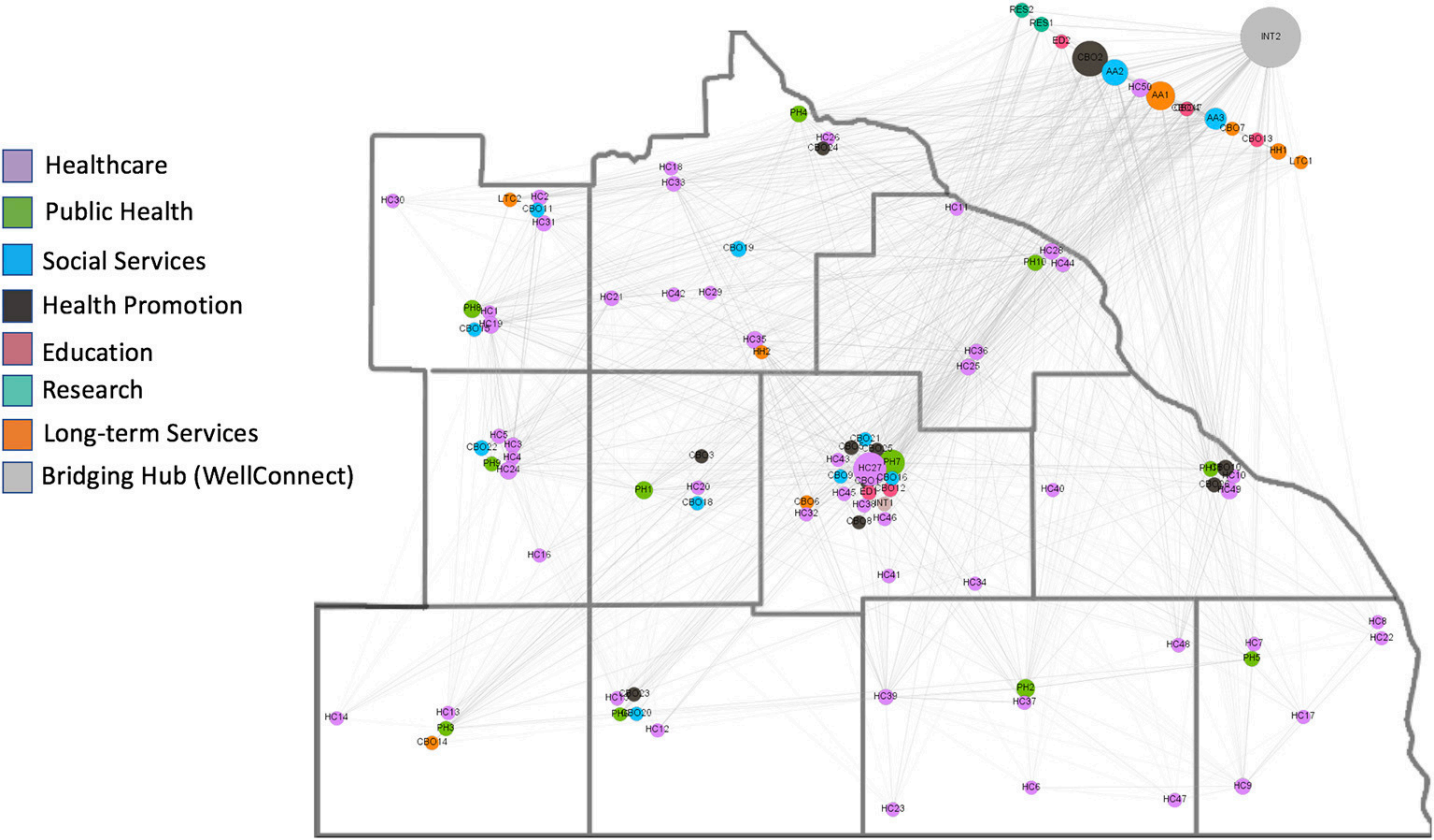
Map of the network in which organizations (small circles) are placed according to their actual locations in the 11 counties. Organizations outside the 11 county region service the entire region. The colors of the circles are representative of the organizations' sector (see legend) and the size is representative of its betweenness centrality (larger circles are organizations with greater betweenness centrality). The large gray circle in the upper right corner is the WellConnect Hub.

**Figure 3:**
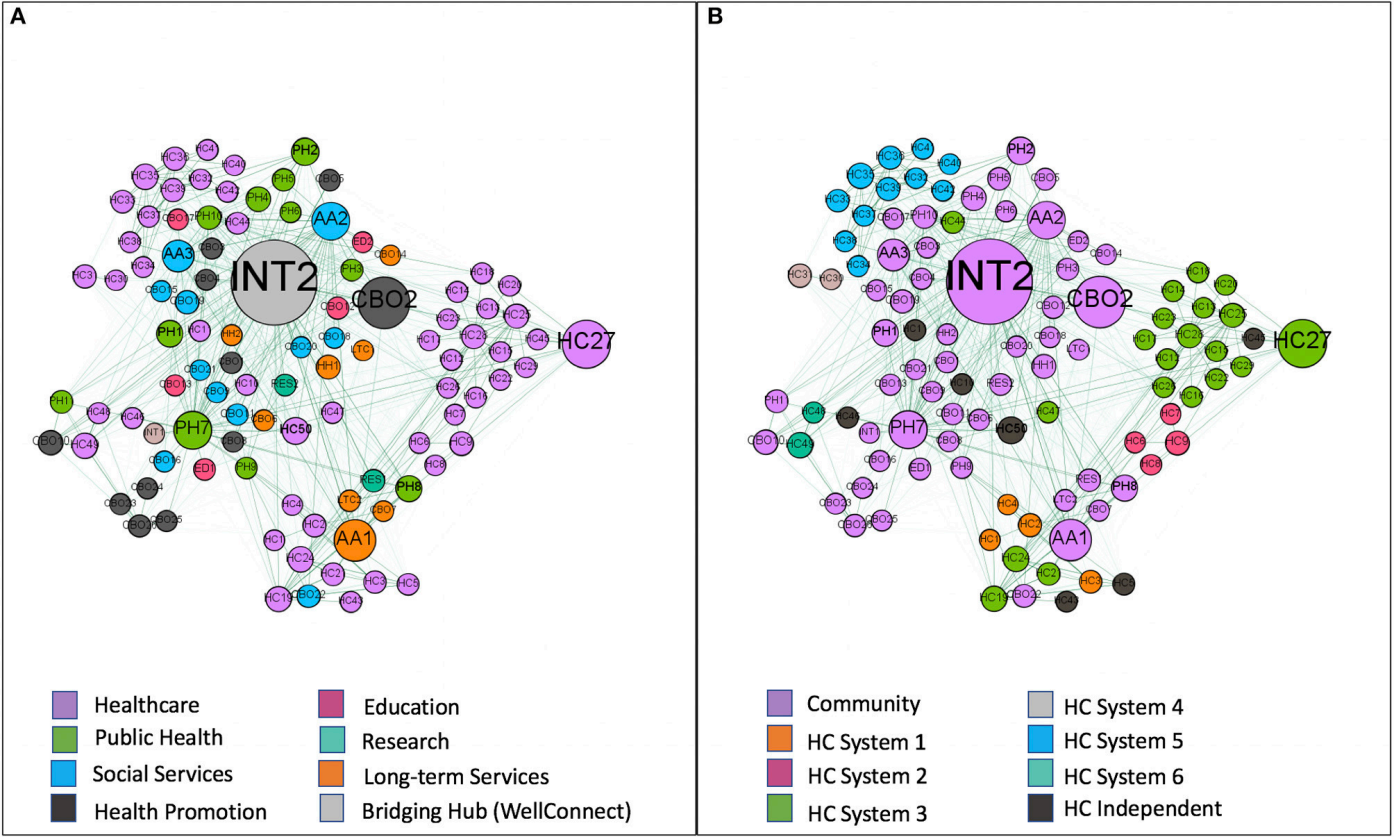
Representations of the network in which organizations (small circles) are positioned by a force-directed algorithm. The size of the circles is representative of the betweenness centrality of the organization (larger circles are organizations with greater betweenness centrality). In image **(A)** the organizations circles are colored by sector (see legend) and in image **(B)** they are colored by whether they are community organizations, part of an integrated healthcare system, or an independent healthcare organization (see legend). The proximity of circles to one another suggests stronger ties. The circle labeled INT2 is the WellConnect hub and both figures show how it is connected most closely to community organizations.

Organizations from multiple sectors were identified in all counties of the region and clustered geographically in ways that were consistent with the distribution of the population (see Figure 2). Identifiable subnetworks separated community organizations from healthcare and healthcare systems from one another (Figure 3). Subnetworks based on geography and sector within the community were present to a lesser extent.

### Role of bridging hub

WellConnect was found to have the highest betweenness (940) and second highest in-degree (34) centrality in the network (Table 3). In general, it appeared that WellConnect had achieved success in becoming a hub for community organizations (it had ties to 39 of 49 surveyed community organizations, 80%) and that it was in good position to bridge healthcare and community organizations and settings. Notably—and consistent with its purpose—WellConnect was connected to all community organizations focused on education, public health, and health promotion (*n* = 28). It was less successful at linking to social and long-term services and supports (9 of 19, 47%). There also remained significant opportunity for improvement in linking healthcare to the community as only 33 of 50 (66%) healthcare organizations were tied to WellConnect. Total hub reach (e.g., the proportion of organizations with ties to WellConnect) was 72% among surveyed organizations and 51% among all organizations identified (e.g., when also including the 38 additional organizations identified by respondents in the denominator—a conservative but reasonable estimate that assumes none of the additional organizations would report a tie to WellConnect).

**Table 3:** Measures of centrality among top 10 organizations in the network.

**In-degree centrality**		**Betweenness centrality**	
**Org ID, description, sector**	**Value**	**Org ID, description, sector**	**Value**
AA1, Area Agency, Social Services	36	INT2, WellConnect, Integrating Hub	940
INT2, WellConnect, Integrating Hub	34	CBO2, Area Charity, Health Promotion	445
CBO17, Area University, Education	33	HC27, Urban Clinic, Health Care	396
CBO2, Area Charity, Health Promotion	29	AA1, Area Agency, Social Services	303
CBO4, Area non-profit, Health Promotion	28	AA2, Area Agency, Social Services	250
AA2, Area Agency, Social Services	27	PH7, Urban County, Public Health	247
PH7, Urban County, Public Health	26	AA3, Area Agency, Social Services	159
HC27, Urban Clinic, Health Care	25	PH2, Rural County, Public Health	98
PH8, Populous Rural County, Public Health	23	HC50, Area Mental Health, Health Care	88
HC38, Urban Clinic, Health Care	23	PH1, Rural County, Public Health	82

### Network collaboration

Across the network's 1,383 ties, the average level of collaboration (e.g., the average weight of the ties, or edges) was 2.16 (representing a level of “Cooperation”). Nearly half (666, or 48%) of the ties suggested a level of Networking only, while levels of Cooperation, Coordination, Coalition, and Collaboration were seen in 21, 11, 6, and 14% of cases, respectively. Perceptions of dyadic collaboration were not consistently matched between organizations in absolute terms (e.g., one organization might score a “3” while the other scores a “5”), although they often represented similar relative judgements (e.g., scores for other organizations might have been mostly “1” in the former case and mostly “3” in the latter).

## Discussion

### Our findings

We described a network-based model to efficiently bridge community-based health promotion and clinic-based health care activities across multiple sites and organizations within a defined geographic region and used SNA to evaluate its implementation shortly after the *de novo* introduction of a bridging hub organization. Consistent with our impressions from earlier research, we found subnetworks defined by an organization's location within the community or within the healthcare system. Healthcare organizations were further divided by integrated system affiliation. The WellConnect bridging hub was in good position to connect healthcare organizations to the health promotion sector, although much opportunity for improvement exists. To that end, the SNA we conducted provided a strong baseline understanding of where efforts could and should be targeted and how progress could be tracked and reported over time.

### Value of methods

This is important as rigorous and actionable evaluations of implementation processes require appreciation of the denominators at play and the social and behavioral relationships that make change happen. Too often, efforts aimed at evaluating multi-organization collaborations describe only the structure and processes of the existing system, without consideration of the potential impact gap, the missed opportunities, and factors that can be influenced to move forward. In that respect, the value of the SNA methods for our purposes cannot be understated. Specifically, we identified 38 organizations that we were previously unaware of and that could play a meaningful role in improving health in the region (especially in the more rural areas and among underserved populations). Three of these organizations were mentioned by multiple organizations and are thus ideal targets for engagement efforts in the network going forward. We also found opportunities to strengthen ties between the bridging hub and some sectors, as well as a need to build capacity in the southwest corner of the region. Lastly, we identified key opportunities to build trust and strengthen ties among some organizations and sectors. These goals can be reported to stakeholders and tracked over time through longitudinal assessments of the measures proposed in Table 1 and with inclusion of the additional organizations identified by respondents.

### Usefulness of the survey

Related to this, the survey measure we used was straightforward to administer and could be feasibly repeated with minimal resources to track progress. Still, we encountered challenges in obtaining a high response rate and saw evidence suggesting the survey response items may have limitations in the population we sampled. For example, despite our efforts to provide respondents with standards for response categories, it seems likely that respondents may perceive the levels to mean different things or that they may have different thresholds for rating at the extremes of the scale. Furthermore, we think it may be challenging for organizations to rate their level of collaboration with another organization on the whole. For example, WellConnect was predisposed to rate its level of collaboration with organizations that sit on its steering committee highly and “as part of one system” because from its perspective and for its purpose that is the case. Surveys sent to these same member organizations were likely to be completed by individuals that do not sit on the steering committee and were predisposed to consider the organization's relationship with the collaborative as a whole and in proportion to all the other things the organization does. These issues did not affect the fundamental insights that were of major practical interest to our purposes, but they may limit the ability of this approach to obtain generalizable knowledge or to reliably track changes over time.

### Recommendations for practice, policy, and research

For all the aforementioned reasons, we have no reservations in recommending the routine use of our SNA methods to collaboratives aiming to build and use a hub organization and that want a better understanding of their environment and the extent to which relevant organizations are aware of and working with the hub. The results generated by these methods may also be useful to policymakers and funders who often find themselves in the position of wanting to support and evaluate the impact of equitable population health initiatives that are focused on an entire geographic region. For these stakeholders to use SNA to track implementation progress and guide decision-making, however, consideration should be given to better calibrating respondents and/or pursuing more resource intensive methods that track and observe behaviors, include in-person interviews, and choose to separate evaluations of organizational interaction and trust. Researchers could take the lead in this area. Specifically, it would be helpful to develop standardized SNA metrics and methods to accompany the implementation of policy-relevant population health models. To the extent these can be adopted by independent and external evaluators, such an effort could facilitate the establishment of best practices and the incentivizing of idealized models.

## Conclusions

In summary, our experience suggests that models depending on complete awareness and use of a bridging hub organization by all relevant organizations within a geographic region are unlikely to be realized overnight. Rather, efforts to develop comprehensive organizational networks mediated by a single coordinating hub involve a process of ongoing learning and targeted implementation. In our case, we identified a set of SNA-based outcome measures that could be used to track progress toward this goal. We found the measures and methods useful and support their adoption in similar situations.

## Availability of data and material

The dataset analyzed during the current study is available in **Additional File 1**.

## Author contributions

AL conceptualized the study and oversaw all aspects of measure development, data collection, and analysis. He developed the first draft of the manuscript and incorporated the feedback of all coauthors before approving the final manuscript. JO conducted the social network analyses and led in the creation of the graphs. She also provided expert guidance on the choice of SNA methods and assisted with drafting the analysis portion of the manuscript. PO assisted with the conduct of the initial literature review and with the drafting of the background and conclusion of the manuscript. She reviewed multiple drafts and approved the final draft. AT assisted with the conduct of the initial literature review and with the distribution and collection of surveys and the entering of survey data. She reviewed multiple drafts of the manuscript and confirmed the accuracy of the data before approving the final draft. FB-F assisted with the literature review and played a key role in conceptualizing the bridging hub model. He reviewed multiple versions of the manuscript and provided feedback before confirming the final version. MW provided significant feedback and edits to multiple versions of the manuscript before approving the final version. RM and RB provided significant feedback and edits to later versions of the manuscript before confirming the final version. VM provided guidance to the study in early stages, reviewed later versions of the manuscript, and provided feedback before approving the final version. All authors approved the final version of the manuscript as submitted.

### Conflict of interest statement

The authors declare that the research was conducted in the absence of any commercial or financial relationships that could be construed as a potential conflict of interest.

## Abbreviations

SNA: social network analysis
CMS: Centers for Medicare and Medicaid Services
CMMI: CMS Innovation Center
AHC: Accountable Health Communities Model
HUB: Pathways Community Hub Model
AHRQ: Agency for Healthcare Research and Quality
ACL: Administration for Community Living
SE MN: Southeast Minnesota.
